# Between allopatry and secondary contact: differentiation and hybridization among three sympatric *Gentiana* species in the Qinghai-Tibet Plateau

**DOI:** 10.1186/s12870-022-03879-0

**Published:** 2022-10-28

**Authors:** Pengcheng Fu, Adrien Favre, Rui Wang, Yizhuo Huang, Shanshan Sun

**Affiliations:** 1 School of Life Science, Luoyang Normal University, 6 Jiqing Road, 471934 Luoyang, P. R. China; 2 Regional nature park of the Trient Valley, La Place 24, 1922 Salvan, Switzerland

**Keywords:** Climatic preference, Divergence, Genomic SNPs, *Gentiana*, Hybridization

## Abstract

**Background:**

Mountains of the world host a significant portion of all terrestrial biodiversity, and the region of the Qinghai-Tibet Plateau (QTP) stands as one of the most remarkable mountain regions on Earth.  Because many explosive radiations occurred there, the QTP is a natural laboratory which is ideal to investigate patterns and processes linked to speciation and diversification. Indeed, understanding how closely related and sympatric species diverged is vital to explore drivers fostering speciation, a topic only rarely investigated in the QTP. By combining genomic and environmental data, we explored the speciation process among three closely related and sympatric species, *Gentiana hexaphylla*, *G. lawrencei* and *G. veitchiorum* in the QTP region.

**Results:**

Combining genome sizes and cytological data, our results showed that *G. hexaphylla* and *G. veitchiorum* are diploid, whereas *G. lawrencei* is tetraploid. Genetic clustering and phylogenetic reconstruction based on genomic SNPs indicated a clear divergence among the three species. Bayesian clustering, migrant, and *D*-statistic analyses all showed an obvious signature of hybridization among the three species, in particular between *G. lawrencei* and both *G. hexaphylla* and *G. veitchiorum* in almost all populations. Environmental variables related to precipitation and particularly temperature showed significant differences among the three gentians, and in fact a redundancy analysis confirmed that temperature and precipitation were the major climatic factors explaining the genetic differentiation among the three species.

**Conclusion:**

Our study suggested that ancient hybridization, polyploidization, geological isolation and the evolution of different climatic preferences were all likely to be involved in the divergence of the three *Gentiana* species, as may be the case for many other taxa in the QTP region.

**Supplementary Information:**

The online version contains supplementary material available at 10.1186/s12870-022-03879-0.

## Introduction

Several major mountain systems of the world are remarkable centres of species diversity, and many of them are classified as hotpots of biodiversity [1–4]. The accumulation of biodiversity in mountains, over evolutionary times, is usually associated with geological processes (e.g., uplift, erosion) and climatic changes (e.g., climate fluctuations) because they combine to generate a complex topography on which a plethora of highly heterogeneous environments co-exist along altitudinal gradients [2, 5, 6]. This heterogeneity fosters for example divergent natural selection and adaptive radiation [7, 8], which were shown to partly explain the uneven distribution of biodiversity in species richness on Earth [9, 10]. Furthermore, diversification may be promoted by climate-driven cycles modifications of distribution ranges, via a so-called species pump effect [3]. Indeed, depending on the climate state (e.g., Last Inter Glacial (LIG) or Last Glacial Maximum (LGM)), mountain ranges may act either as barriers or facilitators of dispersal, causing distribution ranges of organisms to alternate between fragmentation (isolation; allopatric speciation) [2, 11] and expansion (leading to secondary contact; hybridization) [12, 13].

The highest and largest mountain region of the world, the Qinghai-Tibet Plateau (QTP) region, which includes the QTP platform, the Himalayas and particularly the Hengduan Mountains (HM) [14], harbours a rich and probably old alpine flora [15]. The HM, also known as the hotspot of biodiversity “Mountains of Southwest China”, are characterized by deep valleys and a warm and seasonally wet climate [16, 17], while the QTP platform is uniformly high and less rugged with a dry climate. It has been showed that the QTP is home to different species assemblages - or motifs - than the HM [18]. Each species of these assemblages is expected to react individually to climate modifications according to its own ecological preferences. Therefore, numerous case studies are needed to unveil and document the most common phylogeographical patterns as well as processes leading up to speciation and diversification in this topographically and climatically dynamic area. Several studies have tackled the phylogeography of a suite of QTP organisms (e.g., [19, 20]), but this necessary work is still ongoing (e.g., reviewed in Muellner-Riehl [6]).

Geological and climatic dynamics are viewed as major factors fostering intra-specific divergence, speciation and ultimately diversification [14, 21], and only little is known about how biological processes (e.g., adaptation to environment, hybridization) contributed to shape biodiversity in the highly heterogenous environment of the QTP region. For example, in *Circaeaster agrestis*, isolation both by distance and by local adaptation (via adaptive loci related to stress resistance) have been found [20], and in *Pinus densata*, isolation-by-environment explained a significant portion of the genetic structure of the species [22]. These two examples highlight the role of environmental heterogeneity in shaping genetic structure. It was also shown that the molecular signatures of adaptive divergence were similar across two closely related genera in Betulaceae [23], hinting at a parallel effect of habitat heterogeneity on the evolution of reproductive isolation and speciation. Furthermore, a few studies pointed out the crucial role of hybridization in shaping the flora of the QTP region [24], but overall, its extent is probably vastly underestimated there [25]. For example, in the genus *Saxifraga*, the occurrence of hybridization has been reported numerous times in all European clades, whereas it remains almost undetected in the QTP region [26] despite the much larger number of species in the latter area.

**Fig. 1:**
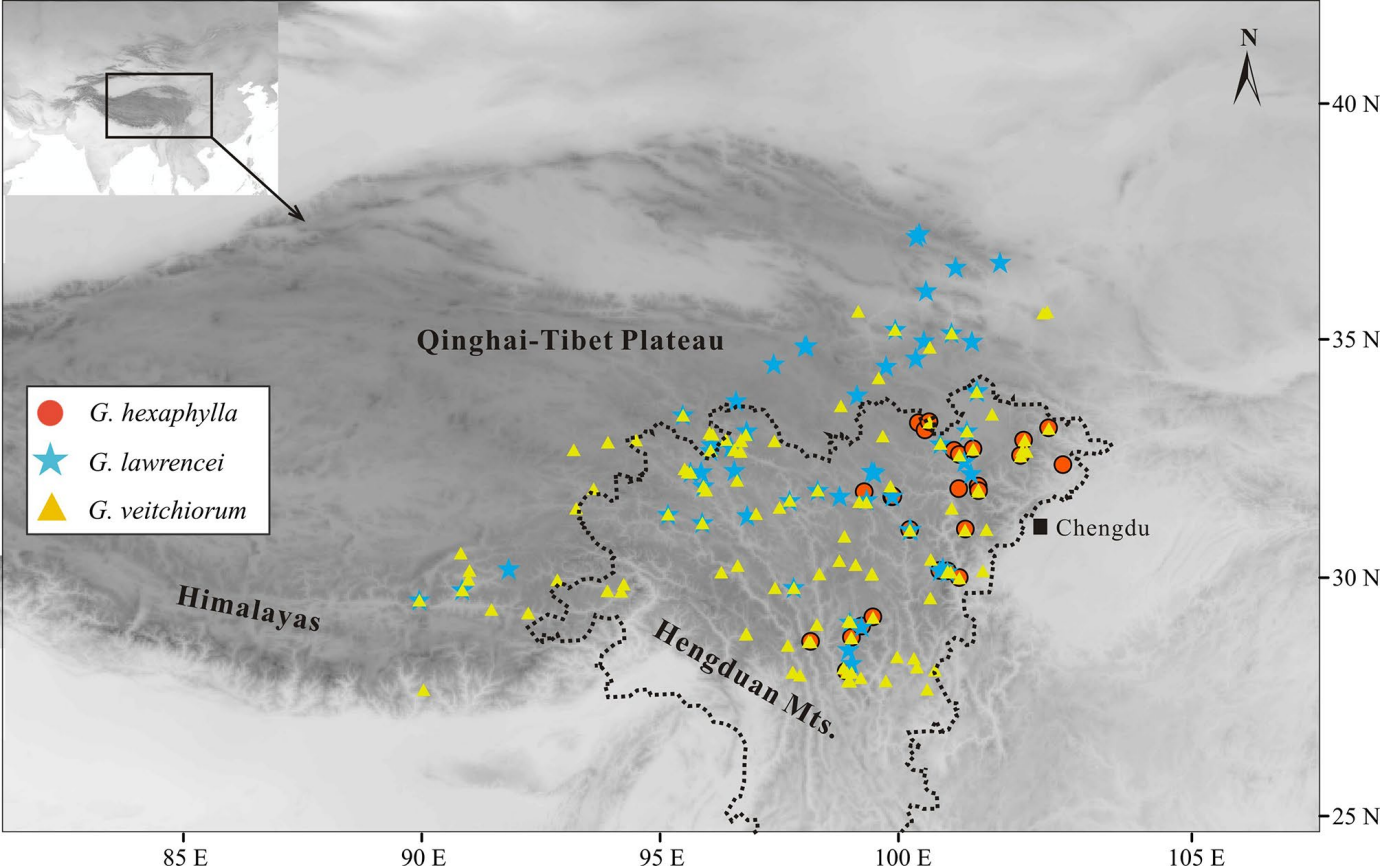
Distribution ranges of three *Gentiana* species based on locality data retrieved from GBIF and fieldwork by the authors (e.g., Fu et al. [19])

The alpine biome of the QTP region is the centre of diversity and the biogeographical origin of many species-rich taxa, including *Gentiana* (Gentianaceae) which is distributed in almost all temperate areas of the world [27, 28]. *Gentiana* is composed of 13 sections [29], some of which being endemic or near endemic to the region of the QTP. This is for example the case of *G.* section *Kudoa* (Masam.) Satake & Toyok. ex Toyok with 25 out of 26 species occurring there [27, 29]. Section *Kudoa* continuously attracted biologists’ attention due to its horticultural and medicinal value [27], its taxonomic complexity [29] and its relevance for evolutionary studies [30]. Previous phylogenetic studies showed a relatively rapid diversification of section *Kudoa* since the Pliocene [30, 31]. Although ploidy levels are not known for all species in this section, five of them are known to be diploids and two are tetraploids [32–35]. In this study, we focused on three closely related species in *G.* section *Kudoa*, namely *G. hexaphylla* Maxim., *G. veitchiorum* Hemsl. and *G. lawrencei* Burkill of which distribution ranges are largely sympatric (Fig. 1)[27]. The three gentians are used in traditional Chinese and Tibetan Medicine and domesticated for horticultural use. The three species can be distinguished by morphological traits such as the shape and arrangement of their leaves (e.g., opposite or in whorls), and the color and shape of their corolla [36]. All three species are perennials and characterized by little pre-zygotic isolation with most visitations being from generalists such as bumblebees [37; personal observations]. Spatial genetic structures were investigated in all three species, unveiling a north-western and south-eastern clade in both *G. veitchiorum* and *G. lawrencei* [19], as well as a northern and southern clade in *G. hexaphylla* [38]. These spatial genetic structures at least partly derive from a combination of climate-driven range displacement and geological barriers [19, 38], but it remains unclear whether their respective environmental preferences also contributed to the genetic patterns observed. In addition, at least one clade of *G. lawrencei* is likely to have experienced hybridization with *G. veitchiorum* in one refugium shared between the two species [19]. Indeed, as in *Saxifraga*, hybridization may be more common than previously thought in the QTP region for *Gentiana* [39–41] given the number of cases of hybridizationdetected in Europe [e.g., 25, 42, 43] and the much larger number of closely-related species in the QTP region [27].

Here, to better understand the mechanisms at work in the process of speciation in *Gentiana*, we combined genomic and climatic data to detect the factors that may have contributed to the divergence among *G. hexaphylla*, *G. veitchiorum* and *G. lawrencei* by sampling across known and spatially structured populations. We specifically aimed to answer (1) Did geographical isolation and climate preferences foster differentiation among the three *Gentiana* species? (2) How did hybridization affect their divergence?

## Results

### Genome size

For genome size estimation, four replications were performed for *G. hexaphylla* and *G. veitchiorum*, and three for *G. lawrencei*. The mean values of the genome size of *G. hexaphylla*, *G. veitchiorum* and *G. lawrencei* were 3.18 G, 3.25 G and 5.00 G, respectively. The standard deviation in the three species ranged from 0.058 to 0.100 (Table S1).

### Data preprocessing and SNP calling

Individuals of *G. veitchiorum* and *G. lawrencei* were newly sequenced in this study, and the raw data of 35 individuals of *G. hexaphylla* were retrieved from Fu et al. [38]. After quality filtering, the number of reads retained per sample varied from 4.45 × 10^6^ to 3.18 × 10^7^, with a median value of 1.04 × 10^7^ (Table S2). The depth per sample varied from 5.76 × to 26.97 ×, averaged at 12.53 ×. After filtering for MAF, linkage-disequilibrium (LD) and missing data, the total number of unlinked SNPs obtained for all samples was 143,611. When the outgroup was included, 144,402 SNPs were kept for downstream analysis.

### Population genetic structure and genetic divergence

**Fig. 2:**
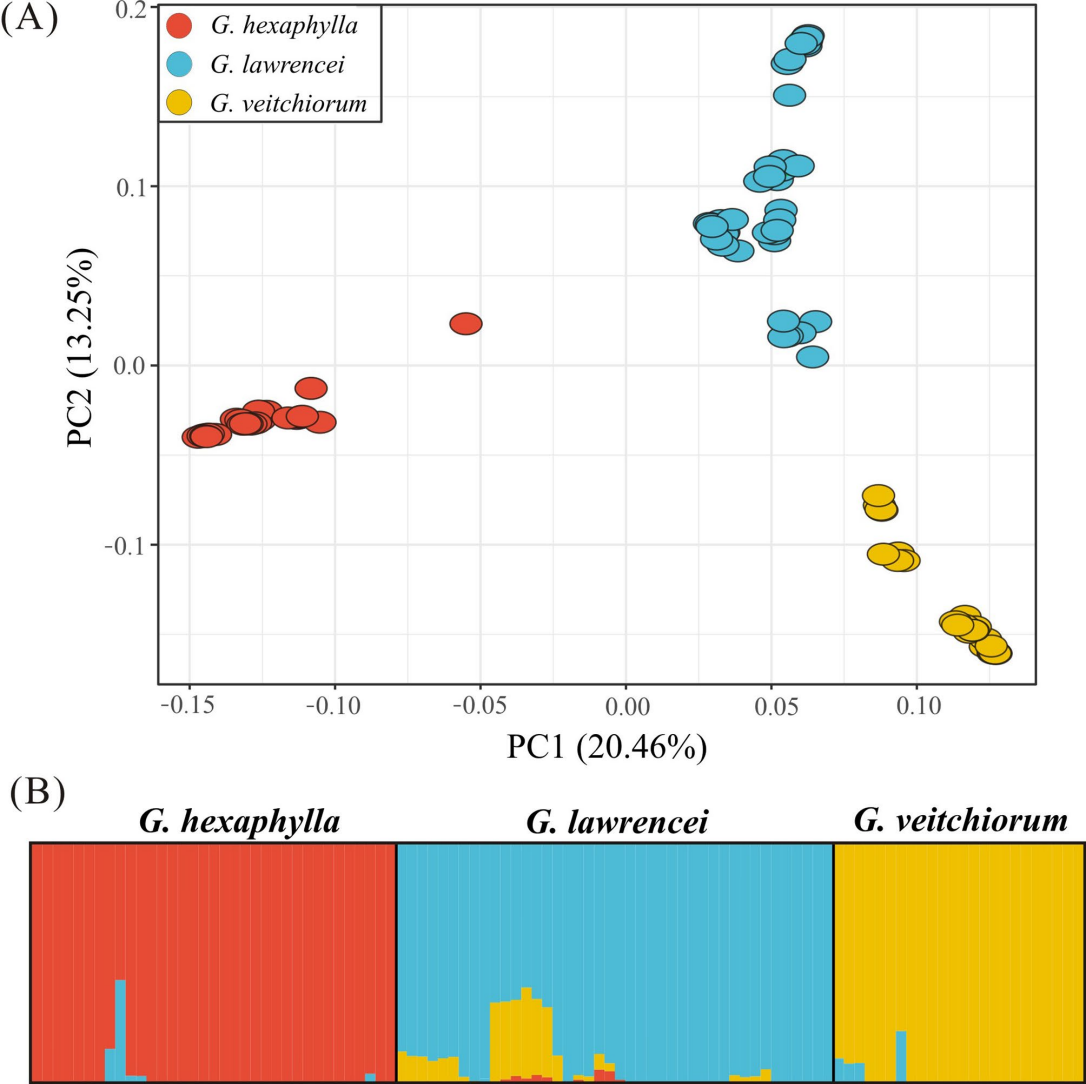
Genetic clustering of three *Gentiana* species based on genomic SNPs. (**A**) Results of principal coordinate analysis. (**B**) Bar plots showing probabilities of ancestral clusters of each sample with K = 3 in Admixture

**Fig. 3:**
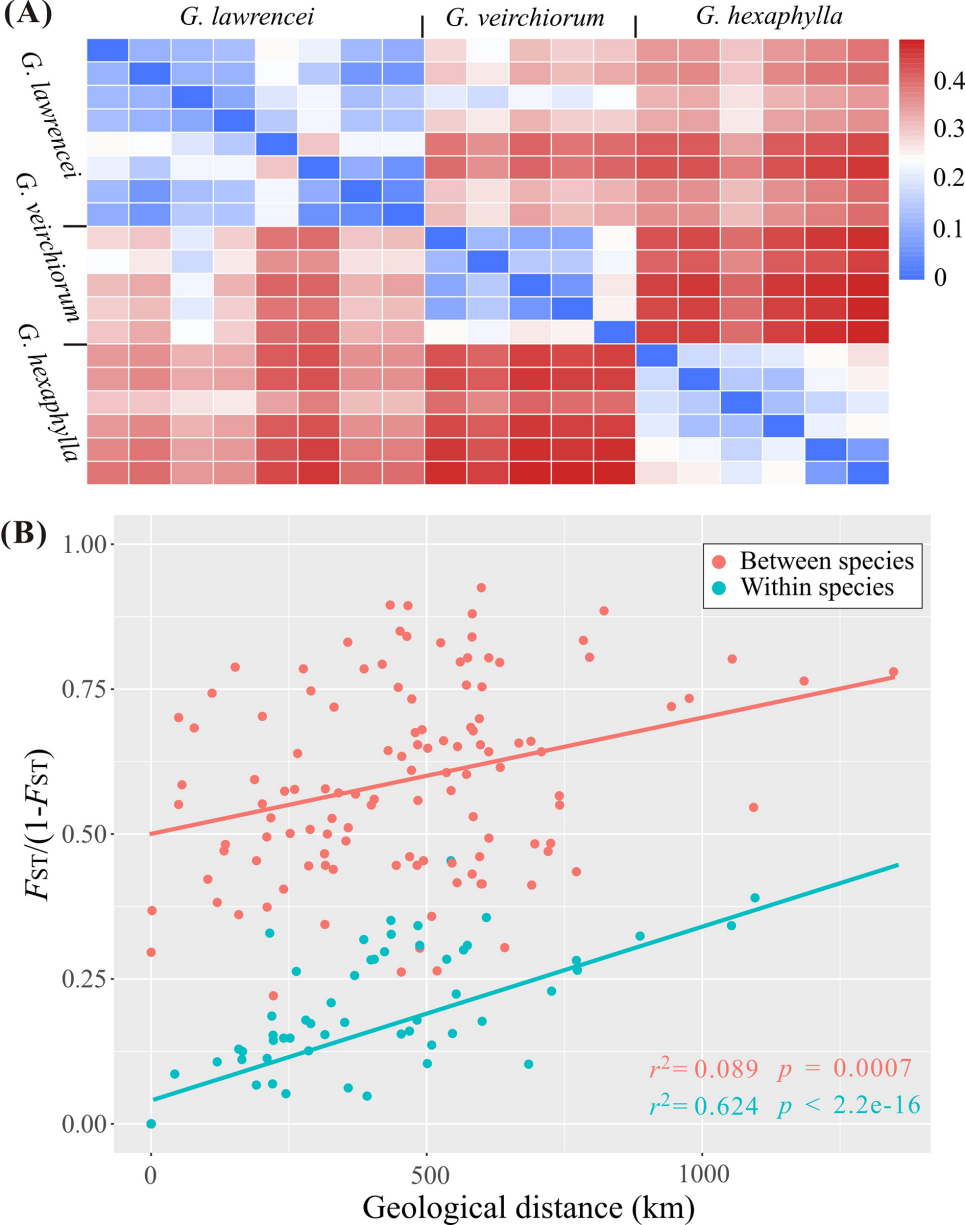
(**A**) Heatmap of weighted Weir and Cockerham’s *F*_ST_; (**B**) Genetic distance (*F*_ST_/(1- *F*_ST_)) against geolographical distance between populations of three *Gentiana* species of section *Kudoa*

Genomic SNPs showed that *G. lawrencei* had a slightly higher genetic diversity (e.g., *A*r, *H*o) than *G. hexaphylla* and *G. veitchiorum* (Table 1). The mean *A*r *vs. H*o in *G. lawrencei*, *G. hexaphylla* and *G. veitchiorum* were 1.1839 vs. 0.1626, 1.1633 vs. 0.1545 and 1.1542 vs. 0.1228, respectively. The Mantel test showed no-significant negetive correlations between altitude and *A*r (*r*^2^ = 0.042, *p* = 0.402) and *H*o_o_ (*r*^2^ = 0.042, *p* = 0.399), respectively. The CV errors from Admixture analyses showed lowest value at K = 3 (Fig. S1), indicating the three species shall be clustering into three groups, so the inferred three genetic clusters corresponded to the three species included in this study (Fig. 2B). Based on the clustering analyses, introgression was detected from *G. lawrencei* to *G. hexaphylla* and *G. veitchiorum*, and from *G. veitchiorum* to *G. lawrencei*. One individual in a population (Fu2016087) of *G. hexaphylla* contained almost equal genetic composition of *G. hexaphylla* and *G. lawrencei*, respectively. From the PCA plot, the first principal component (PC1), which explained 20.46% of all genetic variance, differentiated the three species; the second principal component (PC2), which explained 13.25% of all genetic variance, differentiated the three species as well (Fig. 2 A). Generally, the pairwise *F*_ST_ values were much higher among species than within species (Fig. 3 A). Detailed pairwise *F*_ST_ values between populations were showed in Table S3. The weighted *F*_ST_ values between *G. hexaphylla* and *G. veitchiorum*, *G. hexaphylla* and *G. lawrencei*, and *G. veitchiorum* and *G. lawrencei* were 0.237, 0.187, 0.149, respectively. Plotting *F*_ST_/(1-*F*_ST_) and geographic distances among populations in the three *Gentiana* species showed a significant positive correlation in both within species (*r*^2^ = 0.624, *p* < 2.2e^− 16^) and between species (*r*^2^ = 0.089, *p* = 0.0007) (Fig. 3B).

**Table 1:** Genetic statistics for three *Gentiana* species in the region of the Qinghai-Tibet Plateau. Abbreviations: No., sample size; *A*r, allelic richness; *H*s, mean observed gene diversities within population; *H*o, mean observed heterozygosity within population; *F*is, mean inbreeding coefficient. Abbreviations after localities indicates provinces as follows: QH, Qinghai; SC, Sichuan; SX, Shaanxi; T, Tibet; YN, Yunnan

Species	Voucher no.	Location	Latitude (N)	Longitude ( E)	Altitude (m/a.s.l)	No.	*A*r	*H*s	*H*o	*F*is
*G. lawrencei*	Fu2016025	Aba, SC	32.92	101.81	3500	6	1.1917	0.1748	0.1692	0.0319
	Fu2016039	Seda, SC	32.30	100.28	3926	3	1.1882	0.1615	0.1830	-0.1331
	Fu2016070	Daofu, SC	31.00	101.15	3548	6	1.2140	0.1938	0.1983	-0.0236
	Fu2016089	Kangding, SC	30.08	101.80	4224	6	1.1959	0.1777	0.1731	0.0261
	Fu2016158	Xiangcheng, SC	28.82	100.05	4628	4	1.1393	0.1394	0.1274	0.0862
	Fu2017022	Chenduo, QH	33.20	97.48	4422	6	1.1600	0.1516	0.1351	0.1090
	Fu2017076	Nangqian, QH	31.97	96.51	4317	6	1.1913	0.1710	0.1642	0.0395
	Fu2017264	Gande, QH	34.00	99.94	4045	6	1.1912	0.1739	0.1502	0.1363
*G. veitchiorum*	Fu2017300	Zeku, QH	34.86	100.92	4002	5	1.1599	0.1603	0.1224	0.2366
	Fu2016026	Aba, SC	32.92	101.81	3500	4	1.1455	0.1409	0.1271	0.0981
	Fu2017037	Yushu, QH	33.16	96.65	4287	6	1.1554	0.1563	0.1200	0.2319
	Fu2017096	Dingqing, QH	31.33	95.72	3706	4	1.1579	0.1533	0.1337	0.1281
	Fu2016191	Linzhi, T	29.62	94.67	4434	5	1.1523	0.1562	0.1107	0.2908
*G. hexaphylla*	Fu2019001	Taiba, SX	33.96	107.97	3520	6	1.1511	0.1460	0.1240	0.1508
	Fu2017202	Hongyuan, SC	32.65	102.23	3731	6	1.1655	0.1552	0.1647	-0.0609
	Fu2016087	Kangding, SC	30.07	101.78	4224	6	1.1876	0.1714	0.1784	-0.0406
	Fu2016046	Seda, SC	31.82	100.10	4483	6	1.1637	0.1536	0.1562	-0.0171
	Fu2018052	Deqin, YN	28.33	99.07	4326	6	1.1592	0.1498	0.1557	-0.0390
	Fu2018064	Gongshan, YN	28.07	98.75	3900	6	1.1526	0.1480	0.1479	0.0007

### Phylogenetic relationship and hybridization among species

**Fig. 4:**
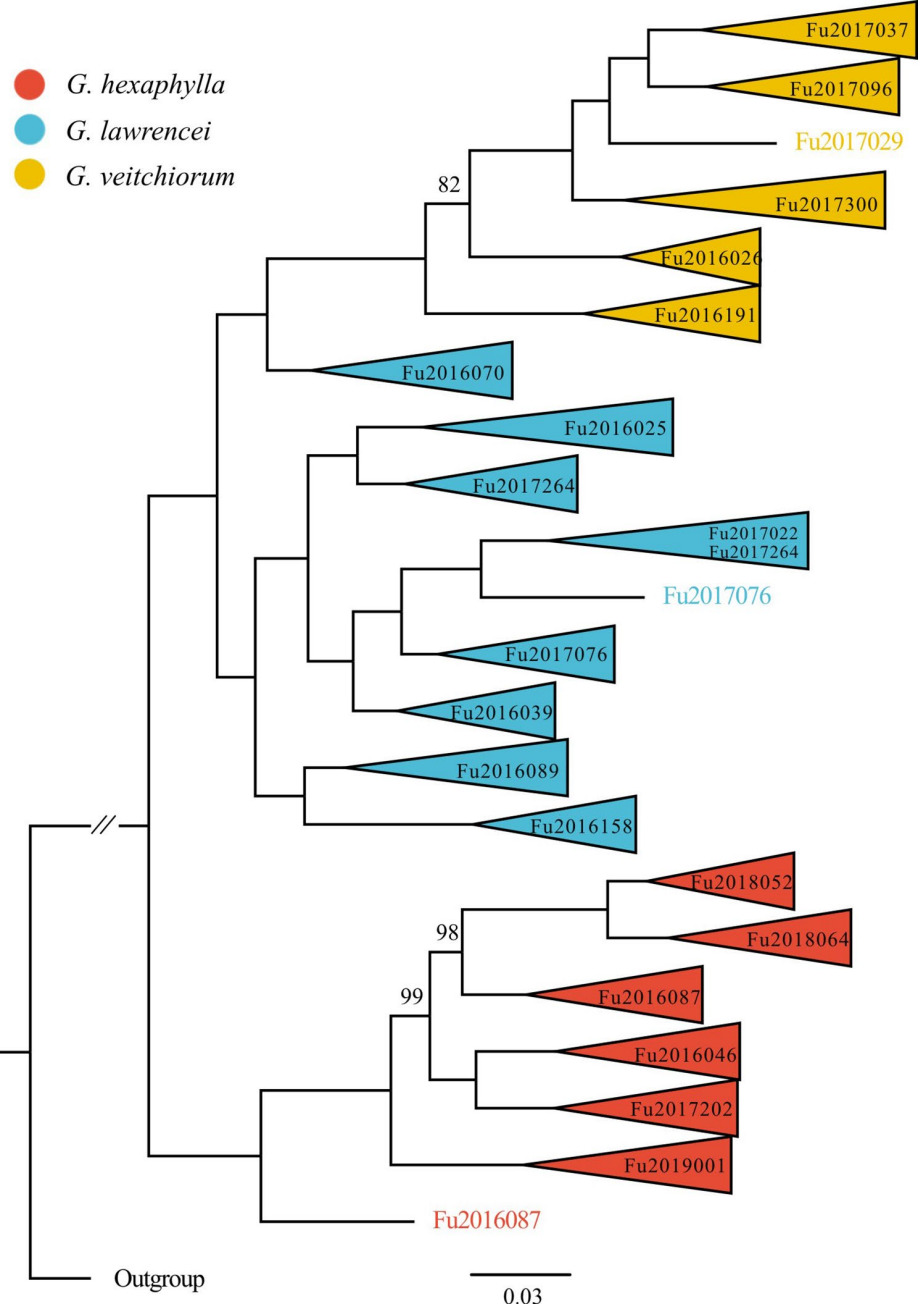
Maximum likelihood tree of three *Gentiana* species based on genomic SNPs. Phylogenetic support values for maximum likelihood were shown above branches only when they differ from 100% bootstrap support. Codes in the tips indicated the population names

Genomic SNPs data resulted in a well-supported tree (Fig. 4). In general, samples clustered together according to the species they were attributed to, except for one population (Fu2016070) of *G. lawrencei*, which was sister to *G. veitchiorum. Gentiana hexaphylla* diverged first, and thus was the sister lineages to both *G. lawrencei* and *G. veitchiorum* (Fig. 4).

**Fig. 5:**
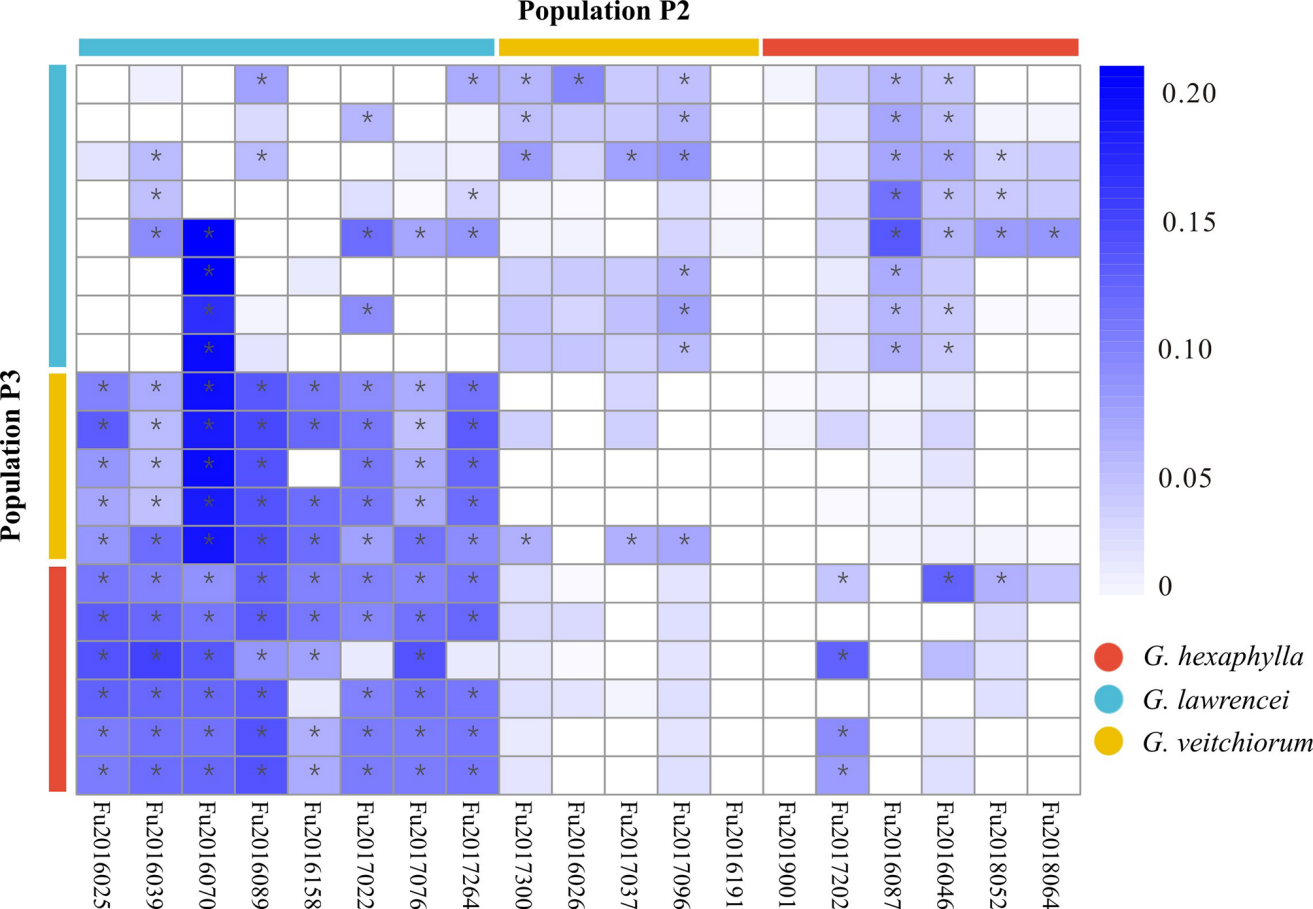
Gene flow detected in the three *Gentiana* species. Heatmap summarizing the *D*-statistic (*D*_BBAA_) estimates and their *P*-values. Taxa P2 and P3 are displayed on the x- and y-axes. Each square represents the highest estimate of each combination of P2 and P3 population. The colour of each square signifies the *D*-statistic estimate. *D*-statistic tests for which *p* < 1×10^-6^ (i.e. were below a Bonferroni-adjusted *P*-value threshold of 0.001) are marked with a black asterisk. The white squares in the matrix indicate no data

Patterson’s *D*-statistic revealed strong signals of introgression between *G. lawrencei* and the other two *Gentiana* species (Fig. 5). The *D*_BBAA_ values between *G. lawrencei* and *G. hexaphylla* showed that introgression was detected in most population pairs between the two species with *p* < 1 × 10^− 6^ (below a Bonferroni-adjusted *P*-value threshold of 0.001). The signal of introgression between most population pairs was also detected in populations of *G. lawrencei* and *G. veitchiorum* (Fig. 5). Week signal of introgression was observed between *G. hexaphylla* and *G. veitchiorum* (Fig. 5).

### Relationship between genomic differentiation and environmental variables

**Fig. 6:**
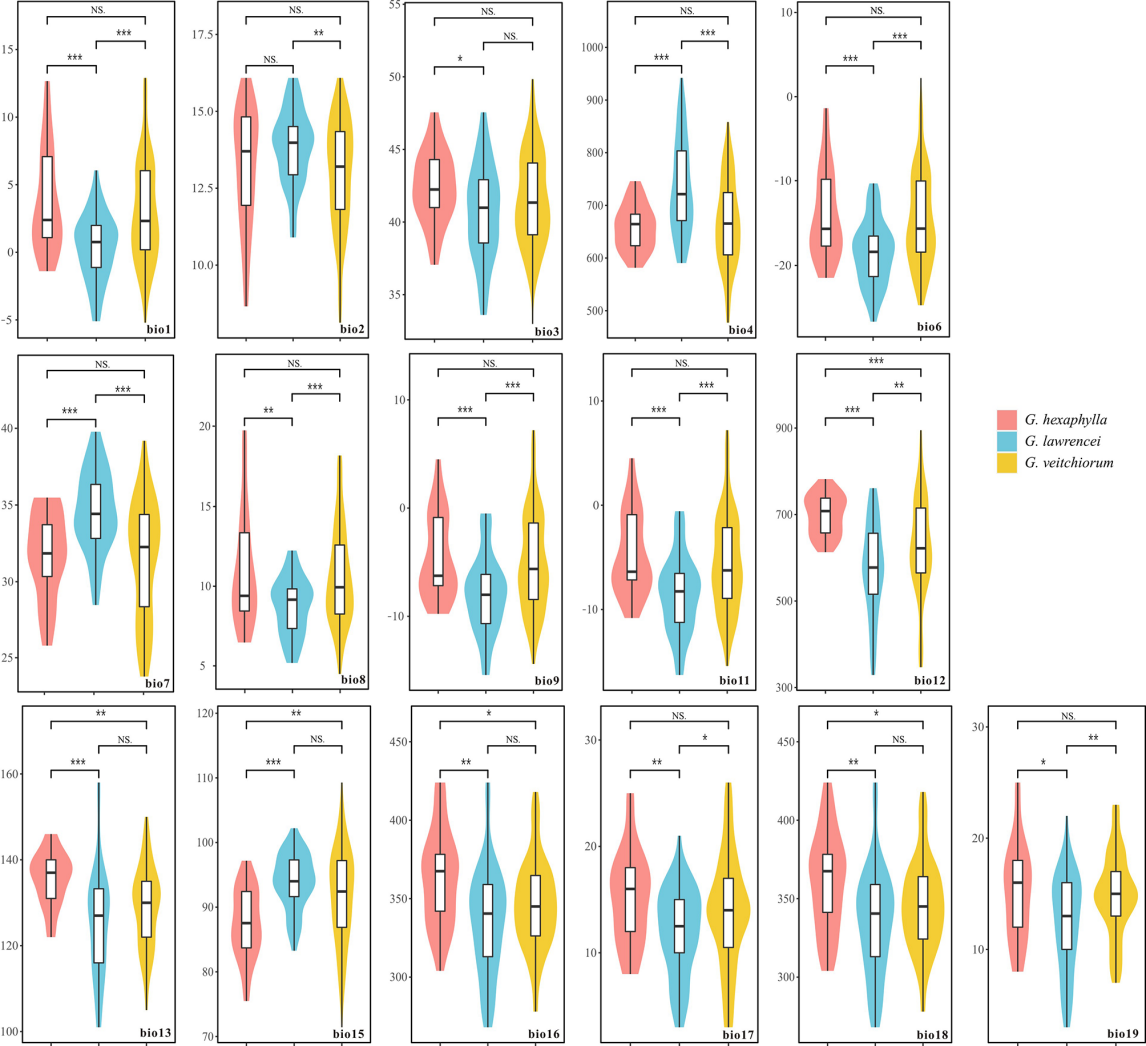
Comparison of climate variables among three *Gentiana* species. Climate variables had no significant difference were not shown. ***, *P* < 0.001; **, *P* < 0.01; *, *P* < 0.05; NS, no significant. Bio1, annual mean temperature; Bio2, Mean Diurnal Range; Bio3, isothermality; Bio4, Temperature Seasonality; Bio6, min temperature of coldest month; Bio7, Temperature Annual Range; Bio8, Mean Temperature of Wettest Quarter; Bio9, Mean Temperature of Driest Quarter; Bio11, Mean Temperature of Coldest Quarter; Bio12, annual precipitation; Bio13, Precipitation of Wettest Month; Bio15, Precipitation Seasonality; Bio16, Precipitation of Wettest Quarter; Bio17, Precipitation of Driest Quarter; Bio18, Precipitation of Warmest Quarter; Bio19, Precipitation of Coldest Quarter

When all the 19 climate variables were analyzed, 16 out of 19 variables (except bio5, bio10 and bio14) showed significant differences between at least two of the three gentians (Fig. 6). The 16 variables could be grouped into two categories related to temperature (bio1–bio4, bio6-bio9, bio11) and precipitation (bio12–bio13, bio15–bio19). *Gentiana lawrencei* showed significant differences with the other two gentians in bio1, bio4, bio6–bio9, bio11, bio12, bio17 and bio19. One variable (bio12; Annual Precipitation) showed significant difference among the three gentians, namely *G. hexaphylla*, *G. veitchiorum* and *G. lawrencei* preferred higher, intermediate, and relatively lower annual mean precipitation, respectively (Fig. 6).

**Fig. 7:**
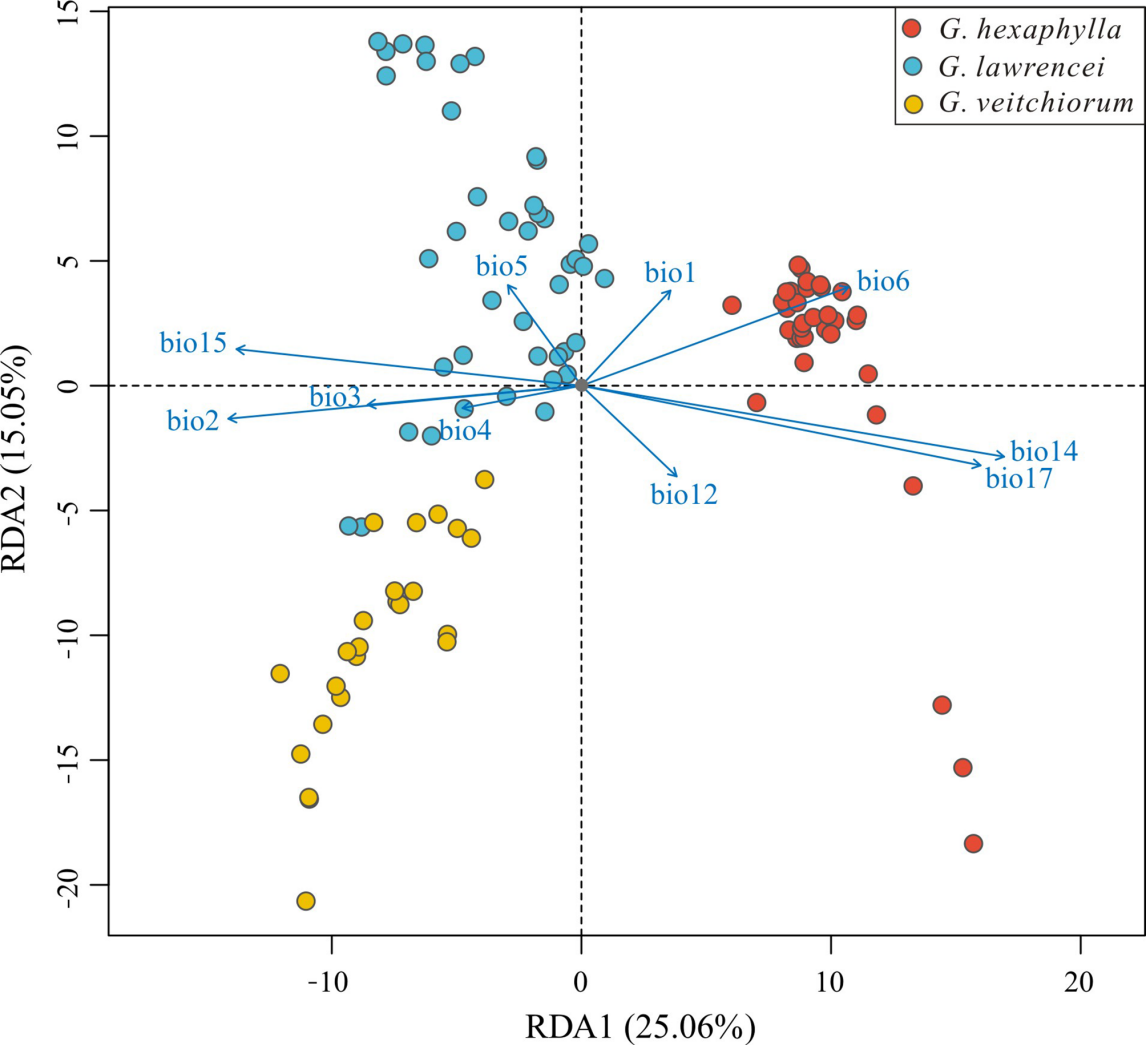
Redundancy analysis showed the relationship between the independent climate variables and genetic divergence among three *Gentiana* species. The colored points represented individuals of the three species

After the Pearson correlation analysis, 10 climatic variables (bio1-bio6, bio12, bio14, bio15, bio17) were kept for the analysis. The results of RDA analysis showed that the 10 combined bioclimatic variables had variables contribution in the first two principal components (Fig. 7). Seven variables, bio2 (Mean Diurnal Range), bio3 (Isothermality), bio4 (Temperature Seasonality), bio6 (Min Temperature of Coldest Month), bio14 (Precipitation of Driest Month), bio15 (Precipitation Seasonality) and bio17 (Precipitation of Driest Quarter) had a more substantial contribution on the first principal component (PC1, 25.06%), and the remaining variables on the second principal component (PC2, 15.05%). Among the variables, bio3, bio4, bio6, bio12 (Annual Precipitation), bio14, bio15 and bio17 were significantly differed (*P* < 0.05, Table 2). *Gentiana hexaphylla* differed with *G. veitchiorum* and *G. lawrencei* along PC1, and the latter two species differed along PC2. For *G. hexaphylla* and the another two gentians, the variables that explained this differentiation were related to temperature and precipitation in months outside of the growing season, for example bio6 (Min Temperature of Coldest Month), bio14 (Precipitation of Driest Month) and bio17 (Precipitation of Driest Quarter) (Fig. 7). For *G. veitchiorum* and *G. lawrencei*, the variables that explained the differentiation were mainly related to bio5 (Max Temperature of Warmest Month).

**Table 2:** Results of the Redundancy analysis (RDA) based on seven un-related environmental variables

Environmental variables	Constrained proportion (%)	*P*
Bio1	1.20	0.087
Bio2	2.40	0.097
Bio3	1.51	0.011 *
Bio4	1.48	0.009 **
Bio5	1.24	0.051
Bio6	1.94	0.001 ***
Bio12	1.42	0.010 **
Bio14	3.36	0.001 ***
Bio15	2.61	0.001 ***
Bio17	3.23	0.001 ***

Bio1, annual mean temperature; Bio2, Mean Diurnal Range; Bio3, isothermality; Bio4, Temperature Seasonality; Bio5, max temperature of warmest month; Bio6, min temperature of coldest month; Bio12, annual precipitation; Bio14, precipitation of driest month. Bio15, Precipitation Seasonality; Bio17, Precipitation of Driest Quarter. *, *P* < 0.05; **, *P* < 0.01; ***, *P* < 0.001

## Discussion

**Genetic divergence among the three*Gentiana*species**.

By sampling *G. hexaphylla*, *G. veitchiorum* and *G. lawrencei* across populations known to be spatially structured and thus building upon previous studies [19, 38, 44], our genomic data showed clear genetic divergence among the three sympatric gentians (Fig. 2). The three species are distinct genetic entities, as supported by several morphological traits such as opposite or whorl leaves, shape of leaves, corolla and calyx lobes, as well as corolla color [27, 36]. Nevertheless, the differentiation among the three species was not very strong as the *F*_ST_ value between species ranged from 0.149 to 0.243. We observed that the genetic divergence within species was sometimes larger than that between species and was sometimes associated with geographical distances (Fig. 3). Interestingly, a weak but significant positive correlation was detected between interspecific genetic distance and geological distance (Fig. 3B), rather than the expected negative correlation [45, 46]. This shows the impact of geographical scale on interspecific divergence in the three gentians. Our findings suggest that both physical barriers and heterogeneous environments may have caused isolation and strengthened differentiation among the three *Gentiana* species. Indeed, previous studies showed that intra-species genetic geographical patterns in *G. veitchiorum* and *G. lawrencei* were shaped by isolation in a southeastern and a northwestern refugia [19, 44], whereas the pattern in *G. hexaphylla* was mainly shaped by geological features at the center of the HM [38]. Genomic data showed that adaption to heterogeneous environments in the QTP region could have produced a high intraspecific divergence [*F*_ST_=0.89, 20] and a parallel adaptive divergence in a number plants taxa [23]. Therefore, we suggest that geographical isolation as well as adaption to heterogeneous environments could have fostered the differentiation among the three *Gentiana* species, and thus had a profound impact in their divergence.

### Ancient hybridization and polyploidization versus divergence

With a remarkable number of radiations occurring in the alpine biome of the region of the QTP [6], of which many closely related species are sympatric, it is now crucial to gather more evidence on the role of hybridization and introgression for adaptation, speciation and ultimately diversification. Our analyses showed obvious gene flow among the three gentians (Figs. 2 and 5), which should come as no surprise since hybridization appears to be more common than previously thought in *Gentiana*. Indeed, it was observed in at least two regions of the world, including the QTP region [e.g., 38–40] and Europe (listed in Favre et al. [25]), as well as in different sections of *Gentiana* (e.g., sect. *Ciminalis*, sect. *Gentiana*, sect. *Cruciata*, etc.). A more frequent occurrence of hybridization in *Gentiana* would also explain some major challenges encountered in taxonomical work and species identification within the genus, as for example in section *Chondrophyllae*. This is also the case for several taxa of *G.* section *Kudoa*, where continuous values for some morphological diagnostic traits were observed [27, 36]. In this section, hybridization may even occur among more species, in this case suggesting a reticulate evolution in its infancy.

In our study, signatures of gene flow were detected especially between *G. lawrencei* towards both *G. hexaphylla* and *G. veitchiorum* in almost all populations (Fig. 5), indicating that their history of hybridization could date back to the origin of these species. Supporting this fact is for example the higher genetic diversity index in *G. lawrencei*, and the lower *F*_ST_ value between *G. lawrencei* and the other two species. In fact, previous studies have already detected some cases of hybridization between *G. lawrencei* and *G. veitchiorum* in a refugium shared by the two species [19]. Furthermore, species distribution models showed that the three species have had a large overlap of potential distribution through time, which increased since the LIG [19, 38], rendering hybridization and introgression progressively more likely in the last few thousand years.

Hybridization may be followed by allopolyploidization, sometimes setting up the base for the emergence of new taxa, as seen for example in birch trees [47]. Whether this has been the case in section *Kudoa* or elsewhere in the genus is still difficult to verify, but it is possible since 30.43% of *Gentiana* species (28 out of 92) for which karyological data are available are in fact polyploids. For the hybridizing species investigated here, karyological studies showed that *G. veitchiorum* is diploid (2n = 2x = 24, [32, 34]), whereas *G. lawrencei* is tetraploid (2n = 4x = 48, [33, 34]). In this study, we estimated that the respective genome sizes of *G. veitchiorum*, *G. lawrencei* and *G. hexaphylla* are 3.25 G, 5.00 G and 3.18 G. These results thus suggest that *G. hexaphylla* – of which ploidy level is still technically unknown - is likely to be a diploid, possibly with ca. 24 chromosomes. Including *G. hexaphylla*, two out of six species in section *Kudoa* are tetraploids [32–35]. Taking their background of hybridization into account, it is not impossible that allopolyploidization participated in the divergence of the three *Kudoa* species investigated in this study. Yet, a more complete investigation on hybridization and allopolyploidization including all species of that section may be needed.

### Did climatic preferences contribute to divergence?

When comparing climatic preferences of *G. hexaphylla*, *G. veitchiorum* and *G. lawrencei*, we found significant differences regarding some components of precipitation and temperature. For example, *G. hexaphylla* and *G. veitchiorum* appear to occur in areas with more precipitation, higher temperature, and smaller temperature annual range than *G. lawrencei* (Fig. 6). In particular, *G. hexaphylla* prefers habitats with more precipitation than the other two species, consistent with its range limited to the HM, which are characterized by a wetter climate than other areas in the QTP region. Our phylogenetic analysis showed that *G. veitchiorum* and *G. lawrencei* diverged from *G. hexaphylla* (Fig. 4), and that divergence among *G. hexaphylla*, *G. veitchiorum* and *G. lawrencei* occurred in the Pleistocene [31], in parallel with the local climate becoming progressively cooler and drier [15, 48, 49]. These results showed that divergence among the three gentians shall be correlated to the evolution of climate preferences and corresponding habitat shifts under past climate modifications.

When associating environmental variables with genetic structure to evaluate the impact of environmental heterogeneity on genetic divergence in the three gentians, we indeed found association between species divergence and environmental variables. In this study, we found a significant association between genetic variation and temperature variables (bio3, bio4, bio6) as well as precipitation (bio14, bio15, bio17) (Fig. 7; Table 2). The results suggested that temperature and precipitation were the important drivers of genetic variation among *G. hexaphylla*, *G. veitchiorum* and *G. lawrencei*, as detected in several plants in different continents [20, 50, 51]. Due to elevation-dependent warming, high-mountain environments experienced more rapid changes in temperature than environments at lower elevations [52]. Therefore, considering the ongoing climate warming, temperature appears to be a key driver for adaptation of the three gentians in the future. Studies about trees in the QTP region showed that altitude, rather than other environmental factors, was the key factor affecting genetic diversity (e.g. [53, 54]), but it is not the scenario we observe in the three herbaceous gentians, although a limited number of populations were tested in this study. Finally, since our analysis did not include other environmental variables, such as soil characteristics, composition and structure, we cannot rule out that habitat adaptation to these environmental variables played a significant role in promoting genetic divergence in the three gentians, as was probably the case in European gentians [25]. Considering soil characteristics would now be necessary to investigate speciation and diversification further in the QTP region, especially since rudimentary knowledge on edaphic preferences of QTP species remains vastly unavailable in *Gentiana* and many other genera.

## Materials and methods

### Studying species and sampling

We chose six, five and eight populations of *Gentiana hexaphylla*, *G. veitchiorum* and *G. lawrencei*, respectively, to represent their ranges and the main genetic clades found in two earlier studies [19, 38]. In total, this study included 35, 24 and 42 individuals of *G. hexaphylla*, *G. veitchiorum* and *G. lawrencei*, respectively. Information about geographical locations and voucher specimens are list in Table 1. All voucher specimens were deposited in the herbarium of Luoyang Normal University.

### Genome size estimation

We used flow cytometry to estimate the genome size of each of the target species using dry material and following the procedure described in Doležel et al. [55]. We used a LB01 lysis buffer to isolate the nuclei for the dried material. One individual of each species was measured three to four times in order to estimate the genome sizes. The cotton (*Gossypium hirsutum*, TM-1, 2.5 G) or tobacco (*Nicotiana tabacum*, Yanyan 97, 4.5 G) were used as standards.

### Library construction, sequencing and SNP calling

Total genomic DNA was extracted from dry leaves using a Dzup plant genomic DNA extraction kit (Sangon, Shanghai, China). For RAD library construction and sequencing [56], each sample was digested with the restriction enzyme *EcoRI*, followed by ligation, purification and size selection as described in Fu et al. [38]. Paired-end reads 150 bp in length were generated using the Illumina Novaseq 6000 (Tianjin, China). Raw reads were filtered and trimmed with Trimmomatic v0.32 [57] with default parameters to remove adaptor sequences and low-quality reads and sites, and then checked for quality with FastQC v0.11.2.

Since RAD-seq study employing reference-based approaches was recommended [58], we mapped the raw reads against the chromosome-level genome of *Gentiana dahurica* (PRJNA799480; [59]), the closest available genome, with bwa-men v2.2.1 [60], and produced the sequence alignment/map format files with SAMtools v1.9 [61]. We marked the PCR duplications with sambamba v0.8.1 [62], and called variations with freebayes v0.9.21 [63] with default parameters. Only SNPs were retained in vcftools 0.1.13 [64] for downstream analysis. SNPs with a minor allele frequency (MAF, less than 5%) and missing frequency of more than 0.8 among individuals were removed using vcftools v0.1.13 [64]. Linkage-disequilibrium SNP pruning was performed in vcftools to excludes variants from each pair closer than 100 bp. We used PGDSpider v2.1.1.5 [65] to convert the final VCF file into different formats to perform further analyses.

### Genetic diversity and population genetic structure

We computed allelic richness (*A*r), observed heterozygosity (*H*_O_), gene diveristy (*H*_S_), and Wright’s inbreeding coefficient (*F*_IS_) using the R package *hierfstat* [66]. To assess the levels of genetic differentiation among populations, we estimated pairwise *F*_ST_ among populations using the Weir and Cockerham method [67] in R package *hierfstat* [66]. A Mantel test was performed in R v. 4.0.1 [68] to check the correlation between genetic diversity (*A*r and *H*_O_) and altitude in each species. The pairwise *F*_ST_ was graphically displayed with package *pheatmap* (https://cran.r-project.org/web/packages/pheatmap/) using R. We plotted *F*_ST_/(1-*F*_ST_) against pairwise geographic distances among populations to illustrate the range-wide isolation by distance (IBD) pattern in R.

For exploring the genetic clusters, we used a Bayesian clustering method implemented in Admixture [69] based on the identified SNPs, with assumed clusters (K) from 1 to 20. The cross-validation (CV) procedure performed 10-fold CV (--cv = 10). The CV errors were plotted in R to assess the model complexity for the data. Graphical representation of individual cluster assignments was performed using DISTRUCT v1.1 [70]. The same data set was used to perform a principal component analysis (PCA), with ten main principal components (PCs) extracted in PLINK v1.90 [71], and visualized using R.

### Phylogenetic analysis

We constructed a phylogenetic tree based on the genomic SNPs using maximum likelihood (ML) in IQ-TREE v.1.6.8 [72] with 1000 ultrafast bootstraps [73]. The best-fitted substitution model was chosen in ModelFinder v2 [74]. The Python script ‘vcf2phylip’ (https://github.com/edgardomortiz/vcf2phylip) was used to transfer the SNPs data for tree building. *Gentiana waltonii* (specimen no. Fu2020030) was served as the outgroup.

### Hybridization analysis

We tested for introgression among the three *Gentiana* species using Patterson’s *D*-statistic [75]. The *D*-statistic uses asymmetry in gene tree topologies to quantify introgression between either of two lineages which share a common ancestor (P1 and P2) and one other lineage (P3) that diverged from the common ancestor of P1 and P2 earlier. We calculated Patterson’s *D*-statistic for all possible population trios using the *Dtrios* function of Dsuite v0.5.r44 [76] with default parameters. We fixed *G. waltonii* as the outgroup. We assessed significance of each test using 100 jackknife resampling runs, and visualized the *D*-statistic estimates in R.

### Environmental data analysis

We obtained 19 climate variables based on the average values from 1950 to 2000 for *G. hexaphylla*, *G. veitchiorum* and *G. lawrencei* from WorldClim (https://www.worldclim.org) using the R package *raster* [77] at 30 arc-second resolutions. Significant differences among the three species for these environmental factors were visualized in R.

Highly correlated variables (Pearson’s correlation coefficient > 0.8, *p* < 0.01) were detected in SPSS and removed to reduce the number of predictors. We used the function *rda* from the R package *vegan* [78] to perform the redundancy analysis (RDA) in order to identify potential environmental factors driving genomic differentiation. We used the function *anova.cca* from the R package *vegan* to check the significance of each predictor.

## Electronic supplementary material

Below is the link to the electronic supplementary material.

Supplementary Material 1

Supplementary Material 2

## Data Availability

The dataset supporting the conclusions of this article is available in the Figshare (doi: 10.6084/m9.figshare.20445879). The raw sequence data reported in this paper have been deposited in the Genome Sequence Archive [79] in National Genomics Data Center [80], China National Center for Bioinformation/Beijing Institute of Genomics, Chinese Academy of Sciences (GSA: CRA008165) that are publicly accessible at https://ngdc.cncb.ac.cn/gsa.
