# Supplementary material for: Between allopatry and secondary contact: differentiation and hybridization among three sympatric Gentiana species in the Qinghai-Tibet Plateau
Source: BMC Plant Biol. 2022 Oct 28;22:504. doi: 10.1186/s12870-022-03879-0 (PMC9615307; doi:10.1186/s12870-022-03879-0)
Supplement: Supplementary file 1 — Supplementary Material 1 [file 12870_2022_3879_MOESM1_ESM.docx]

Table S1. Genome estimation of three *Gentians* species based on flow cytometry.

| Species | Specimen voucher | Values (G) | | | | Mean (G) | SD |
| --- | --- | --- | --- | --- | --- | --- | --- |
| *G. hexaphylla* | Fu2016027-2 | 3.3 | 3.1 | 3.1 | 3.2 | 3.18 | 0.096 |
| *G. veitchiorum* | Fu2016191-4 | 3.2 | 3.2 | 3.3 | 3.3 | 3.25 | 0.058 |
| *G. lawrencei* | Fu2016022-1 | 5.1 | 5 | 4.9 | --- | 5.00 | 0.100 |

Table S2. Information of RAD-seq raw data produced in this study.

| Species | Individual | No. of reads | Depth |
| --- | --- | --- | --- |
| *G. lawrencei* | fu602501 | 8,414,008 | 11.80 |
|  | fu602502 | 6,837,344 | 10.30 |
|  | fu602503 | 11,546,077 | 14.46 |
|  | fu602504 | 7,617,239 | 11.01 |
|  | fu602505 | 7,508,869 | 10.75 |
|  | fu602506 | 6,607,806 | 10.43 |
|  | fu603901 | 8,942,042 | 12.24 |
|  | fu603903 | 9,457,673 | 12.62 |
|  | fu603904 | 9,901,773 | 12.96 |
|  | fu607001 | 9,594,953 | 13.19 |
|  | fu607002 | 8,829,635 | 12.23 |
|  | fu607003 | 9,084,312 | 12.45 |
|  | fu607004 | 9,005,338 | 12.47 |
|  | fu607005 | 8,137,124 | 11.60 |
|  | fu607006 | 9,670,867 | 13.09 |
|  | fu608901 | 10,146,131 | 13.03 |
|  | fu608902 | 8,987,190 | 12.24 |
|  | fu608903 | 7,467,010 | 10.64 |
|  | fu608904 | 6,187,969 | 9.44 |
|  | fu608905 | 8,298,826 | 11.45 |
|  | fu608906 | 8,222,488 | 11.39 |
|  | fu615801 | 13,343,537 | 15.54 |
|  | fu615803 | 13,221,433 | 15.29 |
|  | fu615804 | 8,064,248 | 11.44 |
|  | fu615805 | 11,282,477 | 13.89 |
|  | fu702201 | 8,054,876 | 11.12 |
|  | fu702202 | 10,693,773 | 13.53 |
|  | fu702203 | 7,830,662 | 11.26 |
|  | fu702204 | 8,259,062 | 11.57 |
|  | fu702205 | 7,671,194 | 11.00 |
|  | fu702206 | 7,898,640 | 11.11 |
|  | fu707601 | 8,876,279 | 12.18 |
|  | fu707602 | 8,426,640 | 11.90 |
|  | fu707603 | 7,756,438 | 11.04 |
|  | fu707604 | 7,963,447 | 11.72 |
|  | fu707606 | 8,096,455 | 11.44 |
|  | fu726401 | 6,870,901 | 10.18 |
|  | fu726402 | 10,399,953 | 13.27 |
|  | fu726403 | 7,377,903 | 10.67 |
|  | fu726404 | 7,630,128 | 10.97 |
|  | fu726405 | 10,693,686 | 13.41 |
|  | fu726406 | 9,060,733 | 11.95 |
| *G. veitchiorum* | fu602603 | 10,180,338 | 12.89 |
|  | fu602604 | 8,881,556 | 12.14 |
|  | fu602605 | 8,800,808 | 12.27 |
|  | fu702903 | 5,103,848 | 9.64 |
|  | fu619101 | 7,444,449 | 10.16 |
|  | fu619102 | 9,362,479 | 12.57 |
|  | fu619103 | 8,367,793 | 11.24 |
|  | fu619105 | 9,641,845 | 11.59 |
|  | fu619106 | 8,728,272 | 11.55 |
|  | fu659206 | 5,103,848 | 7.93 |
|  | fu703701 | 7,936,556 | 10.71 |
|  | fu703702 | 5,167,493 | 8.04 |
|  | fu703703 | 4,835,441 | 7.87 |
|  | fu703704 | 7,731,061 | 10.54 |
|  | fu703705 | 5,307,499 | 8.03 |
|  | fu709601 | 6,431,068 | 8.82 |
|  | fu709602 | 6,189,216 | 8.45 |
|  | fu709604 | 8,049,301 | 10.74 |
|  | fu709606 | 9,784,227 | 11.61 |
|  | fu730001 | 6,899,169 | 9.66 |
|  | fu730002 | 4,891,631 | 7.23 |
|  | fu730004 | 5,397,405 | 7.65 |
|  | fu730005 | 5,892,751 | 8.63 |
|  | fu730006 | 12,759,184 | 5.76 |

Table S3. Weir and Cockerham’s *F*_ST_ between each pair of populations based on genomic SNPs in three gentians.

|  | fu2016025 | fu2016039 | fu2016070 | fu2016089 | fu2016158 | fu2017022 | fu2017076 | fu2017264 | fu2017300 | fu2016026 | fu2017037 | fu2017096 | fu2016191 | fu2019001 | fu2017202 | fu2016087 | fu2016046 | fu2018052 | fu2018064 |
| --- | --- | --- | --- | --- | --- | --- | --- | --- | --- | --- | --- | --- | --- | --- | --- | --- | --- | --- | --- |
| fu2016025 | 0.000 |  |  |  |  |  |  |  |  |  |  |  |  |  |  |  |  |  |  |
| fu2016039 | 0.113 | 0.000 |  |  |  |  |  |  |  |  |  |  |  |  |  |  |  |  |  |
| fu2016070 | 0.123 | 0.110 | 0.000 |  |  |  |  |  |  |  |  |  |  |  |  |  |  |  |  |
| fu2016089 | 0.134 | 0.116 | 0.097 | 0.000 |  |  |  |  |  |  |  |  |  |  |  |  |  |  |  |
| fu2016158 | 0.249 | 0.241 | 0.203 | 0.154 | 0.000 |  |  |  |  |  |  |  |  |  |  |  |  |  |  |
| fu2017022 | 0.222 | 0.153 | 0.230 | 0.225 | 0.311 | 0.000 |  |  |  |  |  |  |  |  |  |  |  |  |  |
| fu2017076 | 0.123 | 0.061 | 0.135 | 0.139 | 0.233 | 0.103 | 0.000 |  |  |  |  |  |  |  |  |  |  |  |  |
| fu2017264 | 0.102 | 0.065 | 0.147 | 0.139 | 0.231 | 0.050 | 0.047 | 0.000 |  |  |  |  |  |  |  |  |  |  |  |
| fu2017300 | 0.292 | 0.311 | 0.208 | 0.301 | 0.396 | 0.401 | 0.310 | 0.321 | 0.000 |  |  |  |  |  |  |  |  |  |  |
| fu2016026 | 0.235 | 0.271 | 0.181 | 0.263 | 0.361 | 0.368 | 0.275 | 0.281 | 0.125 | 0.000 |  |  |  |  |  |  |  |  |  |
| fu2017037 | 0.315 | 0.335 | 0.233 | 0.323 | 0.410 | 0.414 | 0.329 | 0.342 | 0.097 | 0.152 | 0.000 |  |  |  |  |  |  |  |  |
| fu2017096 | 0.299 | 0.316 | 0.210 | 0.300 | 0.397 | 0.399 | 0.307 | 0.321 | 0.096 | 0.152 | 0.067 | 0.000 |  |  |  |  |  |  |  |
| fu2016191 | 0.311 | 0.338 | 0.235 | 0.301 | 0.404 | 0.414 | 0.327 | 0.336 | 0.249 | 0.226 | 0.266 | 0.254 | 0.000 |  |  |  |  |  |  |
| fu2019001 | 0.352 | 0.367 | 0.324 | 0.334 | 0.424 | 0.429 | 0.361 | 0.362 | 0.438 | 0.415 | 0.455 | 0.444 | 0.449 | 0.000 |  |  |  |  |  |
| fu2017202 | 0.359 | 0.376 | 0.332 | 0.341 | 0.424 | 0.433 | 0.370 | 0.370 | 0.446 | 0.419 | 0.461 | 0.451 | 0.454 | 0.184 | 0.000 |  |  |  |  |
| fu2016087 | 0.309 | 0.311 | 0.277 | 0.272 | 0.347 | 0.380 | 0.314 | 0.317 | 0.400 | 0.373 | 0.418 | 0.402 | 0.406 | 0.187 | 0.147 | 0.000 |  |  |  |
| fu2016046 | 0.359 | 0.373 | 0.328 | 0.341 | 0.421 | 0.433 | 0.369 | 0.369 | 0.446 | 0.421 | 0.461 | 0.451 | 0.455 | 0.211 | 0.132 | 0.131 | 0.000 |  |  |
| fu2018052 | 0.379 | 0.392 | 0.341 | 0.352 | 0.430 | 0.449 | 0.384 | 0.385 | 0.461 | 0.437 | 0.474 | 0.464 | 0.468 | 0.253 | 0.229 | 0.174 | 0.223 | 0.000 |  |
| fu2018064 | 0.394 | 0.409 | 0.357 | 0.368 | 0.443 | 0.462 | 0.400 | 0.400 | 0.474 | 0.453 | 0.487 | 0.478 | 0.480 | 0.279 | 0.260 | 0.205 | 0.250 | 0.077 | 0.000 |
