# Supplementary figures and images for: Between allopatry and secondary contact: differentiation and hybridization among three sympatric Gentiana species in the Qinghai-Tibet Plateau
Source: BMC Plant Biol. 2022 Oct 28;22:504. doi: 10.1186/s12870-022-03879-0 (PMC9615307; doi:10.1186/s12870-022-03879-0)

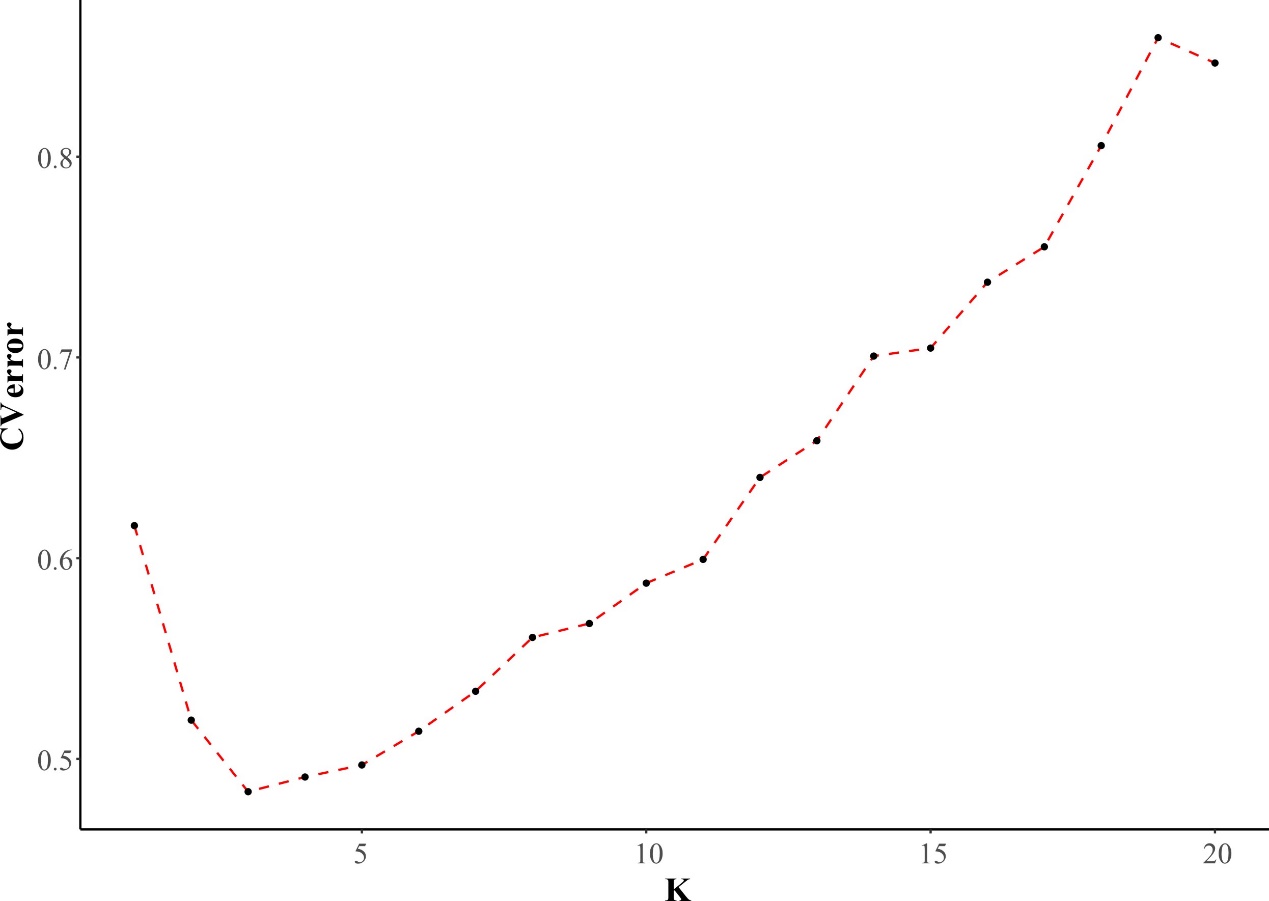


Fig. S1 The cross-validation (CV) error of each K in Admixure.

Supplement: Supplementary file 2 — Supplementary Material 2 [file 12870_2022_3879_MOESM2_ESM.docx]
